# Terahertz scanning of the rabbit cornea with experimental UVB-induced damage: *in vivo* assessment of hydration and its verification

**DOI:** 10.1117/1.JBO.26.4.043010

**Published:** 2021-04-08

**Authors:** Elena N. Iomdina, Sergey V. Seliverstov, Kseniya O. Teplyakova, Elena V. Jani, Viktorya V. Pozdniakova, Olga N. Polyakova, Gregory N. Goltsman

**Affiliations:** a Helmholtz National Medical Research Center of Eye Diseases, Moscow, Russia; b Moscow Pedagogical State University, Department of Physics, Moscow, Russia; c National Research University Higher School of Economics, Moscow Institute of Electronics and Mathematics, Moscow, Russia

**Keywords:** cornea, terahertz radiation, UVB, optical coherent tomography, confocal microscopy, Scheimpflug imaging, rabbit eyes

## Abstract

**Significance:** Water content plays a vital role in the normally functioning visual system; even a minor disruption in the water balance may be harmful. Today, no direct method exists for corneal hydration assessment, while it could be instrumental in early diagnosis and control of a variety of eye diseases. The use of terahertz (THz) radiation, which is highly sensitive to water content, appears to be very promising.

**Aim:** To find out how THz scanning parameters of corneal tissue measured by an experimental setup, specially developed for *in vivo* contactless estimations of corneal reflectivity coefficient (RC), are related to pathological changes in the cornea caused by B-band ultraviolet (UVB) exposure.

**Approach:** The setup was tested on rabbit eyes *in vivo*. Prior to the course of UVB irradiation and 1, 5, and 30 days after it, a series of examinations of the corneal state was made. At the same time points, corneal hydration was assessed by measuring RC.

**Results:** The obtained data confirmed the negative impact of UVB irradiation course on the intensity of tear production and on the corneal thickness and optical parameters. A significant (1.8 times) increase in RC on the 5th day after the irradiation course, followed by a slight decrease on the 30th day after it was revealed. The RC increase measured 5 days after the UVB irradiation course generally corresponded to the increase (by a factor of 1.3) of tear production. RC increase occurred with the corneal edema, which was manifested by corneal thickening (by 18.2% in the middle area and 17.6% in corneal periphery) and an increased volume of corneal tissue (by 17.6%).

**Conclusions:** Our results demonstrate that the proposed approach can be used for *in vivo* contactless estimation of the reflectivity of rabbit cornea in the THz range and, thereby, of cornea hydration.

## 1 Introduction

Water content plays a vital role in the normally functioning visual system; even a minor disruption in the water balance (by 10%) may be harmful.^1^ The majority of water in an organism is bound by connective tissues, which are able to retain water due to the presence of glycosaminoglycans in the intercellular matrix (bound water). The cornea as a connective eye tissue formation normally contains a significant amount (78%) of bound water mass.^2^ If cornea accumulates excessive water (bulk water, causing corneal edema), its refractive properties are changed.^3^ Corneal edema is a characteristic consequence of many eye pathologies, such as endothelial dystrophy, eye condition after cataract surgery or glaucoma filtrating surgery, trauma, ocular toxicity, or hypoxia.^4^^,^^5^ Especially important is the fact that the cornea loses transparency, which leads to significant loss of vision. On the other hand, a dehydrated cornea also changes its shape and refractive ability. If loss of water persists, the cornea is subject to a dystrophic process that causes irreversible disruption of visual function, in particular after eximer laser procedures such as corneal refractive surgeries.^6^^–^^8^ Water content in corneal stroma directly impacts on collagen extracellular matrix and therefore on corneal transparency, i.e., on its optical characteristics.^9^

In view of the above, adequate control and monitoring of corneal hydration is very important for early diagnosis and control of a variety of eye diseases, stating indications for and contraindications to keratorefractive surgeries, and the choice of effective and safe plans of local medication, including tear replacement and hypotensive therapy as well as the choice of contact lens correction regimens.^10^^–^^12^

Currently, corneal edema or dystrophy associated with reduced hydration can only be indirectly diagnosed by measuring the thickness of the cornea as a surrogate parameter for corneal edema (by anterior segment optical coherence tomography or ultrasound pachymetry) or determining the shape of its outer surface (computer keratotopography), as well as determining the biomechanical indices.^13^^,^^14^ However, a change in corneal thickness or shape may not only be associated with an improper hydration, but also with other factors, therefore the existing methods are insufficiently informative in terms of assessing corneal water content.

The use of terahertz (THz) radiation for these purposes is very promising.^15^ At the moment, the THz range is already being used to study biological objects. In particular, the possibilities of this approach are being investigated in the diagnosis of diseases such as breast cancer, skin cancer, as well as in the analysis of wounds, etc.^16^^,^^17^

At present, only a few scientific groups in the world are engaged in the use of THz radiation in ophthalmology. In particular, in Ref. 18, it was demonstrated that THz radiation can be used for quantitative measurement of physiological parameters of the tear film and determination of its hydration. The approach of other authors is based mainly on the use of pulsed THz radiation sources.^19^ For example, in Ref. 20, for scanning the hydration of porcine corneas in the THz range, a traditional scheme consisting of a photoconductive antenna, a femtosecond pump laser, and a Schottky diode as a receiver was used for obtaining pulsed THz radiation. In Ref. 21, the proposed approach was used to obtain images of water content in corneal tissues. However, this method is strongly limited because a powerful and bulky femtosecond laser needs to be used and, therefore, is not suitable for devices intended for clinical practice.

In Ref. 22, it was shown that the transmittance and reflectivity coefficient (RC) of the extracted cornea depend on the water content. In this case, the complex part of the dielectric constant decreases monotonically with a decrease in the water content in the cornea. In their work, the authors used a THz spectrometer with an optimized scanning speed. In another study^23^ by the same authors, the method of time-resolved THz spectroscopy was used for *in vitro* studies of the dependence of the reflection and transmission coefficients of the cornea in the range of 0.1 to 1.5 THz. The results show that the reflection coefficient decreases with increasing radiation frequency, while the absorption coefficient, on the contrary, increases, and it is almost linear. A recent review^24^ of THz methods of non-invasive scanning of corneal hydration for the diagnosis of eye diseases at early stages indicates the growing interest of the international scientific community toward this problem, its relevance, and scientific and clinical importance.

The results of recent studies in the field of THz scanning have demonstrated the effectiveness of this technology for non-invasive determination of the level of hydration of human organs and tissues and its high diagnostic capabilities in various pathological conditions.^25^ Nevertheless, for the best of our knowledge, there are currently no publications on the *in vivo* study of the dynamics of corneal hydration with THz radiation on a well-controlled model of corneal damage, which was the aim of this research.

In our previous work, for the first time in ophthalmology, we have created a test bed for studying corneal hydration using THz scanning, in particular, we have developed a laboratory prototype of the device for monitoring the state of the corneal water balance *in vivo*.^26^^,^^27^ It is necessary to emphasize that THz radiation will interact with both the tear film and the cornea structure. The preliminary data showed that the proposed technology is effective and has the potential for widespread use in clinical ophthalmology.

For further development of THz scanning of the cornea in clinical practice, it was necessary to validate this method on models of ophthalmic pathologies, potentially associated with changes in the water balance of this tissue. As such a pathology, we chose a model of corneal damage caused by ultraviolet (UV) B-range radiation, since it was shown that such an effect leads to an increase in corneal thickness, presumably associated with edema of its stroma.^28^

According the literature data, UVB-induced damage of the eye surface of experimental animals (rabbits) includes loss not only of corneal epithelium, but apoptosis of keratocytes and stromal edema. These changes are accompanied by clinically and histologically manifested corneal inflammation, neutrophil infiltration, nuclear pyknosis, vacuolization of endothelium, and exudation of the anterior chamber of the eye.^29^^,^^30^ Presumably, these changes can be associated with corneal hydration and can impact on the THz RC.

The aim of our study is to find out how THz optical properties of corneal tissue measured by an experimental setup, especially developed for *in vivo* contactless estimations of cornea reflectivity in the THz range, are related to pathological changes in the cornea parameters (thickness, volume, and tear production) caused by B-band ultraviolet (UVB) exposure.

## 2 Experiment

The experimental procedures followed the tenets of the code of ethics of the World Medical Association (Declaration of Helsinki) and were performed according to the 8th edition “Guide for the Care and Use of Laboratory Animals” of the National Research Council and “Statement for the Use of Animals in Ophthalmic and Visual Research” of the Association for Research in Vision and Ophthalmology. The study protocols were approved by the Helmholtz National Medical Research Center of Eye Diseases Ethical Committee.

### 2.1 Material and Methods

The experimental corneal damage modeling was performed according the technique described by Fris et al.^31^ and modified by us. We irradiated 16 eyes of 8 Chinchilla rabbits with 8-W UVB lamp (EB-280C/FE, Spectroline, 312-nm wavelength, 8 W, 230 V, 50 Hz, 0.4 A). The animal’s eyes were irradiated for 4 days during 20 min with a single dose of UVB radiation ($3.12\;{J/\mathrm{cm}}^{2}$) from a distance of 0.4 m. These parameters (in particular, a 20-min exposure of UVB radiation) were used because they were shown to suffice for corneal damage, including the development of corneal edema.^31^ The lamp was located at a normal incidence to the visual axis of the rabbit eye.

**Fig. 1:**
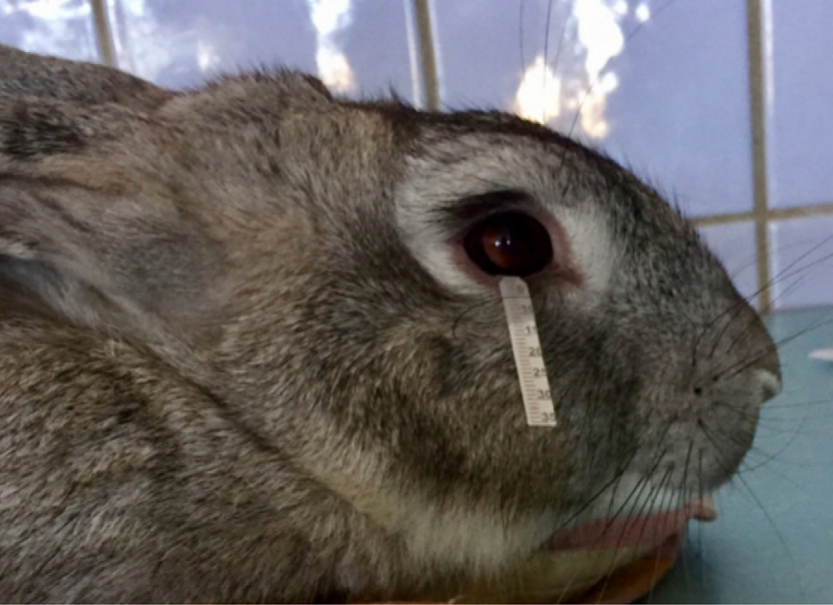
A photo of tear production assessment in rabbit by Schirmer test strip.

Before the course of UVB irradiation and 1, 5, and 30 days after it, tear production evaluation by Schirmer test (Fig. 1) and corneal surface state assessment using fluorescein staining were performed. The complex of examinations of rabbit eyes also included biomicroscopy of corneal epithelium and stroma using slit lamp (XCEL 255), optical coherent tomography of anterior eye segment (OCT AS, Visante TM OCT, version 2.0, model 1000, Zeiss), Scheimpflug visualization and determination of anatomic and optical parameters of anterior eye segment using optical analyzer Galilei G2 (Ziemer Ophthalmic Systems AG 6.0.2), confocal microscopy of the cornea (Confoscan 4, Nidek), and endothelial microscopy (Specular Microscope SP-1P, Topcon, Japan).

THz scanning was carried out before the course of irradiation, as well as 5 and 30 days after the procedure, and included the determination of the RC of the cornea when positioning the continuous THz radiation source and detector at a distance of 1 and 13 cm from the animal’s eye, respectively. The 5th and 30th days were chosen for the estimation of early and late changes of the parameters studied. On the 5th day, the damaging process corresponds to the acute stage of inflammation, and in a month (30th days after UVB irradiation) the inflammation will have for the most part subsided. Thus, we determined the relationship between RC and cornea parameters in these two stages of the process.

**Fig. 2:**
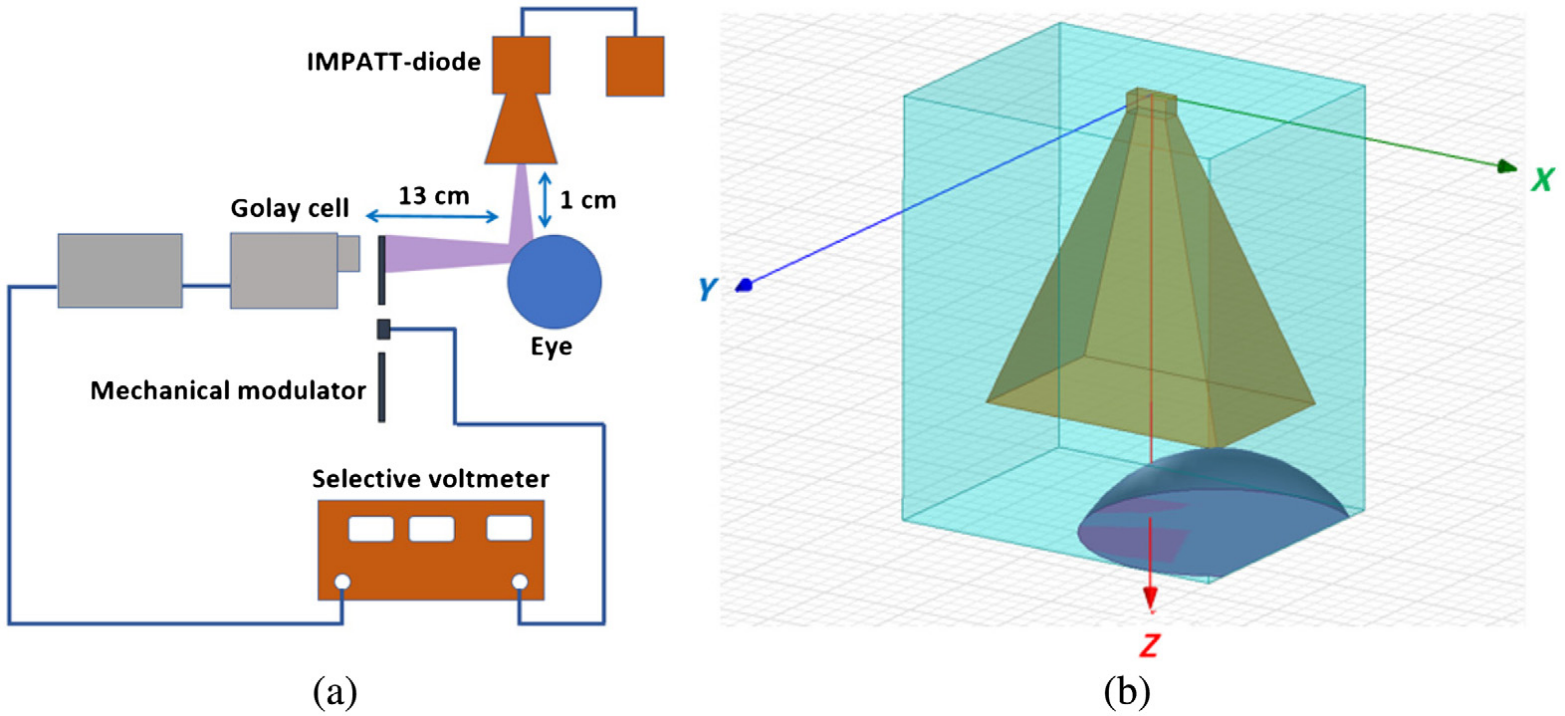
(a) Schematic of an experimental setup for an *in vivo* non-invasive study of the reflectivity of the cornea of rabbits in the THz range. (b) Positioning of the horn antenna in front of the eye.

An impact ionization avalanche transit-time diode (IMPATT) diode with a radiation frequency of 95 GHz was used as a source of THz radiation (see Fig. 2). Radiation from the IMPATT diode with a power of no more than 1 mW was directed to the rabbit cornea using a quasi-optical horn. The power of the reflected scattered radiation was measured using a Golay cell. The experimental setup was preliminary tested in our previous work in the experiment of studying the dependence of the reflection coefficient of the extracted rabbit corneas on the water content in them.^26^ To obtain optimal detector sensitivity, the incident radiation was modulated at a frequency of 10 Hz with a mechanical obturator. The rabbits were immobilized to exclude the impact of eye movements on the reflected signal. The duration of the RC measurement was short, and usually the rabbits’ eyes did not move. If they did move, the results were excluded from consideration. The electrical signal from Golay cell was measured with a selective voltmeter. The magnitude of this signal was used to calculate the reflectivity of the cornea, which is a characteristic of the state of its water balance.

The following scanning parameters were used: the value of the THz signal reflected from a metal plate located at a distance of 40 cm from the detector was 19 to 26 mV; the bias current of the IMPATT diode was 60 mA. All experiments were performed at the same humidity to eliminate the impact of this factor on the eye hydration.

**Fig. 3:**
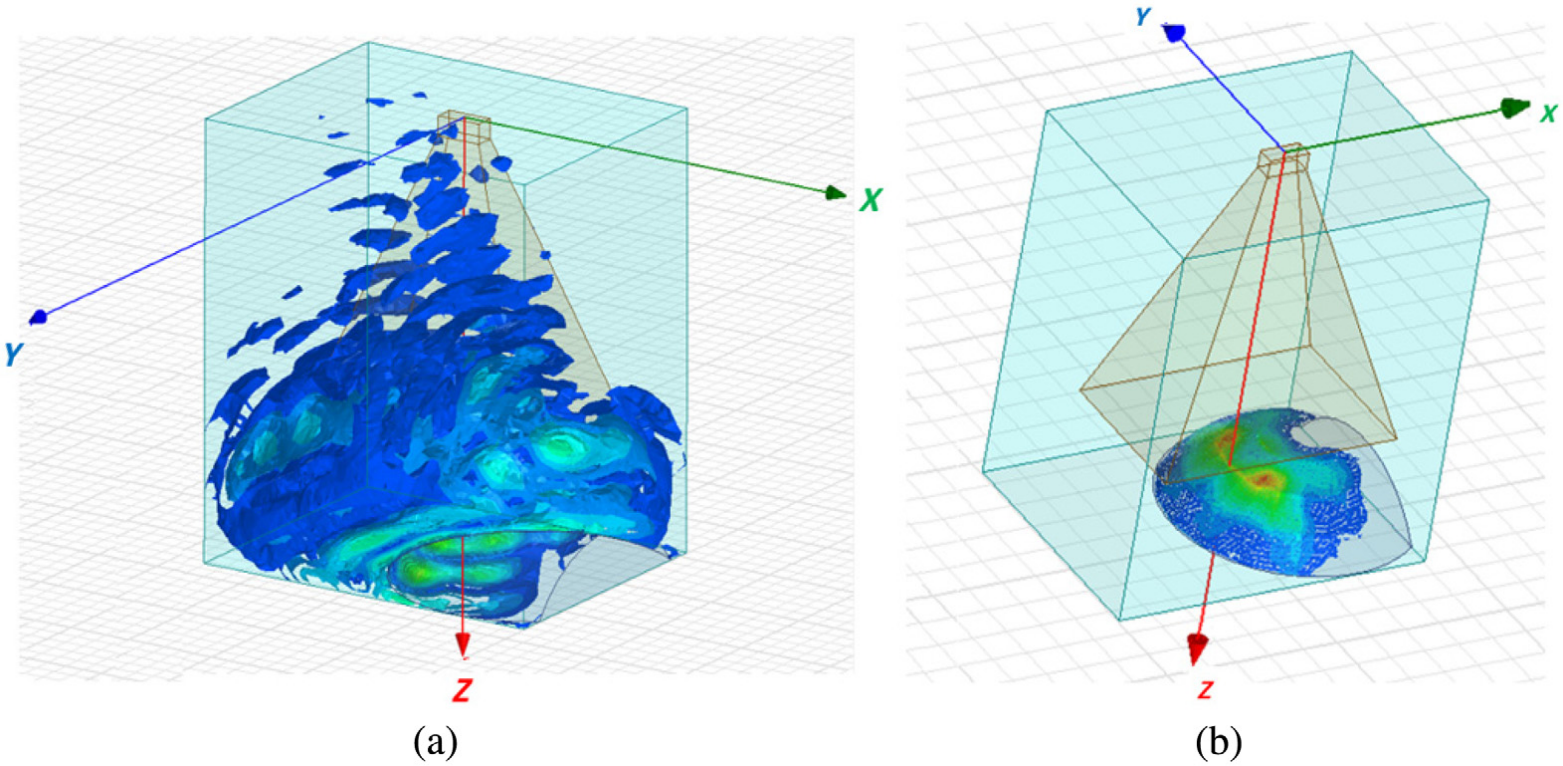
(a) Spatial distribution of the field of incident radiation from the source. (b) Spatial distribution of the SAR of electromagnetic radiation over the surface of the eye.

A 3D electromagnetic simulation was performed using the finite element method. Electromagnetic properties of the cornea (a relative permittivity $\varepsilon_{r}$ of 6.1, a bulk conductivity $\sigma_{\text{bulk}}$ of 57  S/m, and a dielectric loss tangent tan δ of 1.34) at the frequency of 95 GHz were taken from Refs. 18 and 32. The spatial distribution of the incident and reflected radiation field (the magnitude of $\overset{\to }{E}$) with used positioning of the source in front of the rabbit’s eye as well as the distribution of the specific absorption rate (SAR) of radiation over the surface of the eye are shown in Fig. 3. The following equation was used to calculate the SAR: $\sigma E^{2}/(2\rho )$, where σ is the material’s conductivity (defined as $\sigma_{\text{bulk}}+\omega \varepsilon_{0}\varepsilon_{r}\;\tan\;\delta$) and ρ is the mass density of the dielectric material in mass/unit volume. The radiation reflected in the horizontal direction was detected by a Golay cell. An insignificant part of the radiation power in some standing waves occurred in the diagonal direction. The simulation results show that the penetration depth of electromagnetic radiation does not exceed the thickness of the cornea. At the same time, the incident radiation is concentrated in a rather narrow beam, so a possibility of scanning the water concentration over the surface of the cornea and in its stroma is provided.

Statistical processing included nonparametric analysis (medians, quartiles 0.25; 0.75) as well as an assessment of the difference in terms of observation using the Wilcoxon T-test for comparing the results before and after UVB exposure and Spearman’s rank correlation coefficient. The difference was significant if p<0.05.

## 3 Results and Discussion

**Fig. 4:**
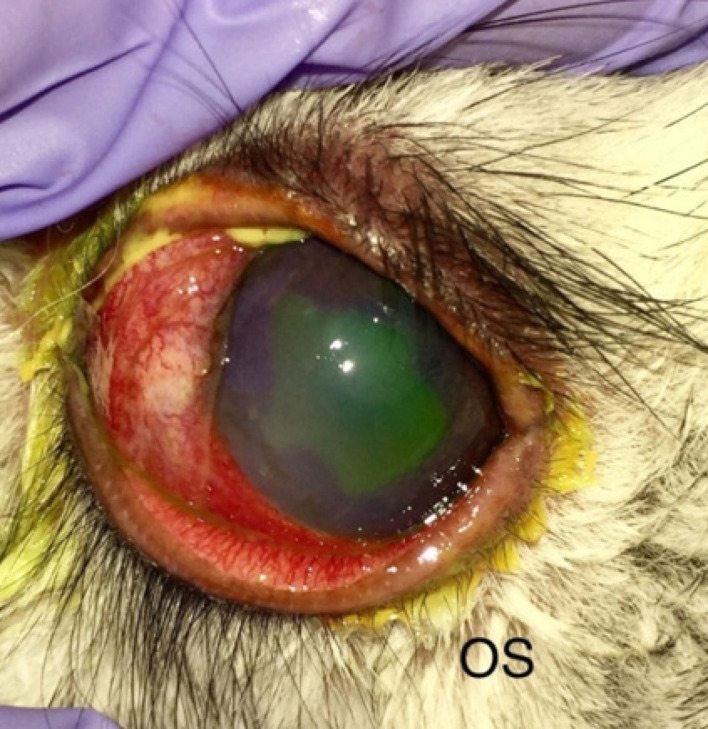
Rabbit eye a day after the end of UVB irradiation course: signs of local inflammation, including conjunctiva and eyelids redness, edema, and defects of epithelium (fluorescein staining).

The results of clinical examination (biomicroscopy) of the rabbit eyes have shown that on the second day the signs of local inflammation were revealed, which included conjunctiva redness and eyelid edemas of various degrees. A day after the end of UVB course, 50% to 100% of corneal surface were covered by defects of epithelium in all experimental eyes (Fig. 4). Five days after the end of UVB irradiation course, sufficient increase of tear production was determined, which was continuously decreasing only by the end of follow-up (Table 1). Confocal microscopy results obtained in 5 days after the end of UVB course demonstrated great amount of desquamated corneal epithelial cells. Endothelial microscopy showed the safety of corneal endothelium after UVB irradiation course.

**Table 1:** Results of tear production assessment of experimental rabbit eyes before and after UVB irradiation course.

Schirmer test result (median [quartile 0.25; quartile 0.75]), mm		
Before irradiation	5 days after irradiation	30 days after irradiation
16.0 [14.0; 18.5]	19.1 [17.5; 21.5]*	14.5 [12.0; 17.5]**

* The difference as compared to the initial level is significant according to Wilcoxon signed-rank test, p<0.05.

** The difference as compared to the initial level is not significant according to Wilcoxon signed-rank test, p>0.5.

Besides this, OCT and Sheimpflug analyzer data (Table 2) showed stromal edema, nonuniform increase of corneal thickness and volume, change of corneal curvature and optical power, as well as a decrease of anterior chamber depth as compared with values of these parameters that we obtained earlier.^33^

**Table 2:** Anatomical and optical parameters of rabbit eye anterior segment before and after UVB irradiation course (median [quartile 0.25; quartile 0.75]).

Parameters	Before irradiation	5 days after
Corneal thickness in middle area, μm	394.1 [175.3; 552.7]	480.3 [262.2; 768.7]*
Corneal thickness in the periphery, μm	396.4 [172.2; 555.0]	489.6 [254.8; 783.4]*
Mean corneal thickness, μm	423.0 [377.2; 486.5]	470.1 [391.8; 564.2]
Corneal tissue volume, ${\mathrm{mm}}^{3}$	20.4 [16.8; 22.6]	24.1 [19.8; 26.9]*
Optical power of the anterior corneal surface in middle area, D	42.7 [41.8; 42.9]	45.6 [42.7; 44.0]*
Curvature radius in the middle area of anterior corneal surface, mm	8.0 [6.9; 8.4]	7.6 [6.1; 8.0]*
Curvature radius in the periphery of anterior corneal surface, mm	8.2 [7.9; 8.4]	7.7 [7.5; 7.8]
Refraction in the cornea apex area, D	44.7 [40.8; 48.7]	48.6 [43.4; 50.7]*
Radius in the cornea apex area, mm	7.6 [7.1; 8.1]	7.0 [6.2; 7.3]*
Eccentricity (asphericity) of posterior cornea surface $e^{2}(-Q)$	1.3 [0.7; 2.1]	4.7 [2.7; 5.2]*
Total corneal refraction in middle area, D	44.1 [39.9; 47.3]	46.0 [42.4; 49.7]*
Anterior chamber depth, mm	3.0 [2.7; 3.1]	2.5 [1.9; 2.8]*

* The difference as compared to the initial level is significant according to Wilcoxon signed-rank test, p<0.5.

Complex of all data obtained confirms the negative impact of UVB irradiation on the intensity of tear production, and on the corneal state, that allows assessing the induced pathology as a suitable model for searching correlations between clinical changes in the tissue and the results of its THz scanning.

THz scanning of the cornea of experimental animals revealed a significant (1.8 times) increase in RC on the 5th day after the irradiation course, followed by a slight decrease on the 30th day after it (amounting to a factor of 1.7 of the initial level), and in all cases the value of this indicator remained significantly higher than the initial level (Table 3).

**Table 3:** Results of THz scan (reflectance) of the cornea of experimental animals before and after UVB irradiation.

Reflected signal (median [quartile 0.25; quartile 0.75]), mV		
Before irradiation	5 days after irradiation	30 days after irradiation
0.78 [0.33; 1.1]	1.2 [0.57; 2.4]*	0.98 [0.50; 1.8]**

* The difference as compared to the initial level is significant according to Wilcoxon signed-rank test, p<0.01.

** The difference as compared to the initial level is significant according to Wilcoxon signed-rank test, p=0.01.

Comparison of obtained results with above-mentioned clinical data gives an evidence to propose the significant correlations between RC changes and the parameters of the course of UVB-induced pathological process. Thus, the RC increase measured 5 days after the UVB irradiation course generally corresponded to the increase (by a factor of 1.3) of tear production (Table 1).

The RC increase (with regard to the initial RC level) 5 days after the UVB exposure course was mostly in moderate inverse relationship with the value of the initial individual parameter of Schirmer test (Spearman’s correlation coefficient r=−0.387). In most cases, more expressed RC increase in 5 days was noted in case of lowest initial tear production and vice versa, in cases of high initial values of Schirmer test the increase of RC was less expressed. Obviously, normal tear production and as a consequence normal tear film better protect a cornea against edema induced by UVB damage.

Important qualitative relationships were revealed when changes of RC and anatomical and optical parameters measured using optical analyzer were compared (Tables 2 and 3). In particular, RC increase occurred with the corneal edema which was manifested by corneal thickening (by 18.2% in the middle area and 17.6% in corneal periphery) and an increased volume of corneal tissue (by 17.6%). The increase of these parameters in 5 days after UVB irradiation course corresponded to the increase of RC in this follow-up period.

At 30 days after UVB exposure, RC remained above baseline and signs of corneal edema were still detected. Although the cornea has become thinner compared to the 5-day period (413.1 [207.2;688.4]  μm in the middle area and 414.3 [210.1;699.8]  μm in the periphery) and more uniform in thickness, but small differences in thickness compared to the initial level (see Table 3) in the middle zone (5%) and on the periphery of the cornea (4.5%) remained. The corneal tissue volume (21.2 $[17.1;23.9]\;{\mathrm{mm}}^{3}$) approached the initial value by this time, but a slight increase (by 4.5%) compared to the initial level remained.

Thus, the *in vivo* verification of THz scanning for the detection of the dynamics of corneal hydration on the model of controlled UVB-induced inflammation and edema of this tissue has been made. To the best of our knowledge, it is the first time when such verification is made.

## 4 Conclusion

Our results demonstrate that the proposed approach can be used for *in vivo* contactless estimation of the reflectivity of rabbit cornea in the THz range and, thereby, of cornea hydration. On the one hand, RC values correspond to the dynamics of certain indirect parameters of corneal edema induced by UVB exposure at different stages of the pathologic process and, on the other hand, an RC value is a more informative parameter because it directly represents the overall corneal hydration. The benefit of determining the reflection coefficient is explained by the fact that this parameter directly characterizes the water content in the cornea. As the changes in the water content are consistent with the qualitative changes of the thickness and volume of the cornea and tear production at certain stages of the pathological process occurring in the cornea, we conclude that the developed experimental setup is valid and informative and can justifiably be used in the overall *in vivo* assessment of water content in the cornea.
